# The Effects of HIV on the Sensitivity of a Whole Blood IFN-γ Release Assay in Zambian Adults with Active Tuberculosis

**DOI:** 10.1371/journal.pone.0002489

**Published:** 2008-06-18

**Authors:** Edward Raby, Maureen Moyo, Akash Devendra, Joseph Banda, Petra De Haas, Helen Ayles, Peter Godfrey-Faussett

**Affiliations:** 1 Department of Infectious and Tropical Diseases, London School of Hygiene and Tropical Medicine, London, United Kingdom; 2 The Zambia AIDS Related TB (ZAMBART) Project, Department of Microbiology, University Teaching Hospital, Lusaka, Zambia; 3 The Zambia AIDS Related TB (ZAMBART) Project, Department of Pathology, University Teaching Hospital, Lusaka, Zambia; 4 The Zambia AIDS Related TB (ZAMBART) Project, Department of Medicine, University Teaching Hospital, Lusaka, Zambia; McGill University, Canada

## Abstract

**Background:**

Interferon gamma release assays (IGRA) are replacing the tuberculin skin test (TST) as a diagnostic tool for Mycobacterium tuberculosis infection. However research into the test's performance in the high HIV-TB burden setting is scarce. This study aimed to define the sensitivity of an IGRA, QuantiFERON-TB® Gold In-Tube (QGIT), in adult Zambian patients with active smear-positive tuberculosis. Secondary outcomes focussed on the effect of HIV on the test's performance.

**Principal Findings:**

Patients attending government health clinics were recruited within 1 month of starting treatment for TB. Subjects were tested with QGIT and TST. T lymphocyte counts were estimated (CD3^+^, CD4^+^, CD8^+^). QGIT was performed for 112 subjects. 83/112 were QGIT positive giving an overall sensitivity of 74% [95%CI: 66,82]. A marked decrease in sensitivity was observed in HIV positive patients with 37/59 (63%) being QGIT positive compared to 31/37 (84%) HIV negative patients [chi^2^ p = 0.033]. Low CD4^+^ count was associated with increases in both indeterminate and false-negative results. Low CD4^+^ count in combination with high/normal CD8^+^ count was associated with false-negative results. TST was recorded for 92 patients, 62/92 were positive, giving a sensitivity of 67% [95%CI: 58,77]. Although there was little difference in the overall sensitivities, agreement between TST and QGIT was poor.

**Conclusions:**

QGIT was technically feasible with results in HIV negative subjects comparable to those achieved elsewhere. However, where under-treated HIV is prevalent, an increased proportion of both indeterminate and false-negative QGIT results can be expected in patients with active TB. The implications of this for the diagnosis of LTBI by QGIT is unclear. The diagnostic and prognostic relevance of IGRAs in high burden settings needs to be better characterised.

## Introduction

Infection with *Mycobacterium tuberculosis* (MTB) results in nine million new cases of tuberculosis disease (TB) and just under two million deaths a year [1] The vast majority of TB is found in South-East Asia, Africa and the western Pacific regions. However, it is only in the African region and particularly in eastern and southern Africa that the incidence continues to rise. This increase is fuelled by the dual epidemic of TB and HIV and poses considerable challenges for TB control [2]. Strengthening DOTS and improving anti-retroviral therapy (ART) services are essential [3]. Improving detection and treatment of latent tuberculosis infection (LTBI) is also important.

The natural history of TB is altered in the presence of HIV infection. The risk of both primary progressive disease and reactivation of latent tuberculosis infection (LTBI) is increased, resulting in a high incidence of active disease in this population [4], [5]. There is also an increased proportion of smear negative disease [6]. The detection and treatment of LTBI with isoniazid has proven efficacy in reducing incidence of active disease but as a policy is poorly implemented [7], [8]. One barrier to the effective management of LTBI is a lack of accurate diagnostic tools.

MTB is a slowly multiplying, intracellular pathogen that is capable of surviving for many years in an immunocompetent human host. As bacterial load is low in LTBI, conventional microbiology has little to offer to aid diagnosis [9]. The tuberculin skin test (TST) has been the most extensively used immune-based test. A positive TST has clear association with an increased risk of developing active tuberculosis. If used as a screening test, isoniazid preventative therapy has greatest effect in a population who are TST positive [10]. However many factors including infection with non-tuberculous mycobacteria, use of BCG and immunosuppression have been identified as influencing the test outcome [11]. In particular the test has a much lower sensitivity in HIV positive patients [12].

Western medicine has incorporated interferon-gamma release assays (IGRAs) into everyday practice and there is a growing literature characterising their performance in this setting [13]. In contrast, there is a scarcity of data from low income, high burden countries. In the absence of a gold standard LTBI diagnostic, active TB has been used as a surrogate in order to estimate the sensitivity of IGRAs. Only two studies from Africa have been published that use this approach. In a township in Cape Town where between 55 and 61% of TB patients are thought to be HIV infected, 100/154 (65%) culture positive TB patients were QuantiFERON-TB® Gold In-Tube (QGIT) positive [14]. Only 41 patients knew their HIV status, of these, 17/26 (65%) HIV positive subjects had a positive IGRA result compared with 11/15 (73%) HIV negative. Those that were HIV positive had lower responses to TB antigens and all 5 indeterminate QGIT results occurred in the HIV positive group. As part of a larger study in The Gambia, 80 recently diagnosed culture positive TB patients were recruited [15]. 7/80 (8.8%) were HIV positive. The authors report results for 75 subjects of whom 48 were QGIT positive giving a sensitivity of 64.0% (95%CI: 51,73). The only other study from a high burden area reports QGIT results for 60 pulmonary TB patients of whom 97% were culture positive and 5% HIV positive [16]. Their interferon-gamma responses were then followed through the course of TB treatment. Prior to treatment, 44/60 (73%) were QGIT positive. Responses fluctuated over the following six months but with no significant change in overall sensitivity.

In this study, we aimed to define the sensitivity of QGIT in Zambian adults with smear positive TB, focussing on the effect of HIV infection on the test's performance.

## Methods

### Study Population

The study was conducted in the government clinics of the four Zambia and South Africa tuberculosis and AIDS reduction study (ZAMSTAR) sites in Lusaka. In 2004, Zambia reported the sixth highest notification rate in Africa with 471 new or relapsed cases per 100 000 population. The true incidence is estimated to be over 600 per 100 000. HIV prevalence among pregnant women in urban areas is greater than 20%, with an estimated prevalence of HIV among adult TB cases of 54% [17]. The study protocol was approved by London School of Hygiene and Tropical Medicine ethics committee and by the University of Zambia ethical research committee.

### Eligibility & Recruitment

Recruitment occurred over the period of July to October 2007. The target population was all patients recorded as smear positive over the age of 18 within one month of commencing treatment. Patients were defined as smear positive if acid-fast bacilli were detected by direct light microscopy in at least one sputum smear. These patients are required to attend clinic daily for observed therapy. Eligible patients were identified through the review of patient-held treatment cards on arrival at clinic. In exceptional circumstances due to infirmity or work commitments a surrogate collects daily drugs for the patient or a week's supply is given. No efforts were made to trace non-attenders or defaulters, however, we attempted to identify and capture weekly attenders by reviewing clinic-held treatment cards.

### Venepuncture

Venous blood was obtained by needle and syringe and immediately transferred to evacuated blood collection tubes that were inverted 8 times to thoroughly mix contents. Nursing staff in Lusaka had previously reported problems with slow and incomplete filling of QGIT tubes (manufactured by Becton, Dickinson and company - BD, Franklin Lakes, USA, supplied by Cellestis, Carnegie, Australia) using the vacutainer system alone. This is presumably due to the small volume and relatively low negative pressure in the tubes compared to the ambient pressure in Lusaka at around 1300 m above sea level. By using a syringe, correct filling was ensured so that each of the QGIT tubes received 1 ml and the K_2_EDTA tube (BD) for T lymphocyte estimations at least 1 ml. The full set of three QGIT tubes was used comprising one coated with each of TB RD1 test antigens, PHA as a positive control or heparin for the negative control. Blood collection occurred between 08:00 and 12:00 with all QGIT samples placed simultaneously at 12:00 in a portable incubator (Cellestis) preheated to 37°C.

### TST

TST was placed according to the Mantoux method using two tuberculin units PPD RT23 in 0.1 ml Tween-80 (Statens Serum Institut, Copenhagen, Denmark) [18]. Transverse induration was measured in millimetres using callipers on one occasion between 48 and 164 hours after placement. This wide window for reading was used in an attempt to improve capture and has been shown to give reasonably reliable results [19]. In patients with TB and HIV, cutaneous anergy is thought to be an all or nothing effect and so there may be little gain in terms of reducing false-negative TST results by lowering the cut-off from 10 mm to 5 mm [20]. However standard practice and is to use the 5 mm cut-off in HIV positive patients. As all of our subjects had active TB and the majority were expected to be HIV positive, the 5 mm cut-off was used to define a positive TST in the main analysis. A secondary analysis applied the 10 mm cut-off for comparison.

### Laboratory Procedures

T lymphocyte estimations were performed within eight hours of sampling. CD3^+^, CD4^+^ and CD8^+^ were measured by flow cytometry (FACSCount, BD) according to the manufacturer's instructions using standard reagents (BD). On arrival in the laboratory, and after no more than 2 hours from the start of incubation, QGIT samples were transferred to a Jouan incubator (Thermo Fisher Scientific, Waltham, USA) to complete 24 hours at 37°C. Samples were then returned to room temperature and, after no more than 2 hours, centrifuged for 10 mins at 2200 RCF (Thermo). Plasma was extracted and transferred to −20°C. ELISA was performed precisely according to manufacturer's instructions using standard kits (Cellestis). Data acquired was transferred to QuantiFERON-TB® Gold analysis software (Cellestis) for results calculation.

### Analysis of QGIT cut-off value

To calculate QGIT results, a value is calculated for the concentration of IFN-gamma in the TB antigen tube minus the corresponding concentration in the nil tube to take into account background IFN-gamma level. The cut-off value for positive results is set by the manufacturer at ≥0.35 IU/ml. It is possible that this cut-off is not appropriate in all populations. In particular it has been suggested that a lower cut-off may be appropriate in populations where TB is prevalent and in HIV infected patients with reduced T lymphocyte counts [21]. A lower cut-off value was suggested for South Korea by these investigators following receiver operating characteristic (ROC) analysis of their data. Although none of their subjects were HIV positive 29 of 87 were classified as immunocompromised. A study of untreated culture-confirmed cavitary pulmonary tuberculosis patients in Turkey also found that reducing the cut-off to between 0.05 and 0.10 IU/ml improved sensitivity with little effect on specificity [22]. The main analysis for this study uses the manufacturer's suggested cut-off of ≥0.35 IU/ml. A sub-analysis was performed to assess the impact of lowering the cut-off to ≥0.13 IU/ml.

### Data Handling

Data were analysed in STATA v.10 (StataCorp LP, Texas, USA). When calculating sensitivity some authors have been tempted to disregarded indeterminate results labelling them as uninterpretable [14]. This definition artificially inflates the test sensitivity and is less applicable on the population level. Furthermore, if better understood, indeterminate results may also be able to provide important clinical information on a personal level. Sensitivity was therefore defined as number of positive results over total number tested.

## Results

### Subject characteristics

112 adults with smear-positive tuberculosis were recruited (table 1). 71 (63%) were male, median age was 31 (range: 18,58; IQR: 25,36). 40 (36%) had sputum smear recorded as 1+, 18 (16%) as 2+ and 53 (47%) as 3+, 1 (1%) was unknown. 20 (18%) were relapse cases. 30 (27%) had received no treatment, 60 (54%) were in the first two weeks of treatment and the remaining 21 (19%) had had between 2 and four weeks. 96 subjects had HIV status recorded, of these, 59 (61%) were HIV positive. 105 subjects had T-lymphocyte estimations performed. Median CD4^+^ count overall was 316 cells/μl (range: 6,1708; IQR: 170,594), dropping to 212 cells/μl (range: 6,1015; IQR: 109,332) in those recorded as being HIV positive and with a broad range in the HIV negative subjects (median 542; range: 84,1708, IQR: 437,698). Median body mass index (BMI) was 19 (range:13,25; IQR: 17,21).

**Table 1:** Summary demographic and clinical characteristics of subjects.

Variable	Subcategory	n/N (%) or Median (IQR)
Sex	Male	71/112 (63)
Smear grade	1^+^	40/111 (36)
	2^+^	18/111 (16)
	3^+^	53/111 (48)
	Unknown	1/112 (1)
BCG scar	Present	71/109 (65)
	Unknown	3/112 (3)
HIV status	Positive	59/96 (61)
	Unknown	16/112 (14)
ART	Started	14/112 (13)
TB treatment period	None	30/111 (27)
	1 to 14 days	60/111 (54)
	15 to 31 days	21/111 (19)
Relapse case		20/112 (18)
Age, years		31 (25,36)
BMI, kg/m^2^		19 (17,21)
CD3^+^, cells/μl		1112 (808,1687)
CD8^+^, cells/μl		715 (344, 1028)
CD4^+^, cells/μl	Overall	316 (170,594)
	HIV negative	542 (437,698)
	HIV positive	212 (109,332)

ART, antiretroviral therapy; BMI, body mass index.

### QGIT

83/112 were QGIT positive giving an overall sensitivity of 74% (95%CI: 66,82). Of the remainder, 13 (12%) had negative and 16 (14%) indeterminate results (table 2). In subjects recorded as being HIV negative, 31/37 were QGIT positive giving a sensitivity of 84% (95%CI: 71,96). Among the HIV positive subjects, sensitivity was significantly reduced to 63% (95%CI: 50,75), 37/59 being QGIT positive with a marked increase in the proportion of negative results (Pearson's chi^2^, 2df, p = 0.033).

**Figure 1:**
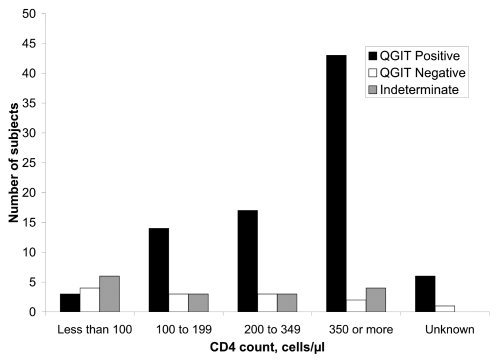
Distribution of QuantiFERON-TB® Gold In-Tube results by CD4^+^ lymphocyte count. Proportion of both negative and indeterminate results increased with falling CD4^+^ lymphocyte count. QGIT, QuantiFERON-TB® Gold In-Tube.

A low CD4^+^ count has previously been shown to reduce the sensitivity of QGIT [5], [23]. To explore this effect, data were stratified according to CD4^+^ count. <100 cells/μl represents a very low CD4^+^ count, <200 is AIDS defining, 350 is the lower end of the normal range for men. With falling CD4^+^ count there was a decrease in sensitivity of QGIT with relative increases in both negative and indeterminate results (Pearson's chi^2^, 6df, p<0.001). This was particularly marked at counts less than 100 cells/μl, where only 3/13 (23%) had a positive QGIT result (figure 1).

**Table 2:** Distribution of QuantiFERON-TB® Gold In-Tube results by HIV status, CD4^+^ lymphocyte count, treatment period and TST result.

		QGIT result, n/N_row_ (%)		
		Positive	Negative	Indeterminate
Overall		83/112 (74)	13/112 (12)	16/112 (14)
HIV Status	Negative	31/37 (84)	1/37 (3)	5/37 (13)
	Positive	37/59 (63)	12/59 (20)	10/59 (17)
	Unknown	15/16 (94)	0/16 (0)	1/16 (6)
CD4^+^ count, cells/μl	Less than 100	3/13 (23)	4/13 (31)	6/13 (46)
	100 to 199	14/20 (70)	3/20 (15)	3/20 (15)
	200 to 349	17/23 (74)	3/23 (13)	3/23 (13)
	350 or more	43/49 (88)	2/49 (4)	4/49 (8)
	Unknown	6/7 (86)	1/7 (14)	0/7 (0)
Treatment period	0 days	21/30 (70)	6/30 (20)	3/30 (10)
	1 to 14 days	46/60 (77)	4/60 (7)	10/60 (16)
	15 to 31 days	15/21 (72)	3/21 (14)	3/21 (14)
TST	≥5 mm	51/62 (82)	6/62 (10)	5/62 (8)
	<5 mm	22/30 (74)	4/30 (13)	4/30 (13)

QGIT, QuantiFERON-TB® Gold In-Tube; TST, Tuberculin Skin Test.

The time on treatment was not seen to alter the proportion of results in each category (Pearson's chi^2^, 2df, p = 0.377; table 2).

For further analysis of factors affecting sensitivity, QGIT result was transformed to a binary variable by combining negative and indeterminate results. In order to increase statistical power, continuous variables were redefined as dichotomous variables using the following arbitrary cut-off values: age 31 years (median of study population), BMI 18.5 kg/m^2^ (World Health Organisation definition of malnutrition in adults), CD4^+^ count 200 cells/μl (AIDS defining), TB treatment period of 2 weeks.

In univariate analysis, a CD4 count greater than 200 cells/μl was the only variable to show a significant association with positive QGIT result and this persisted when adjusted for age, sex, smear status, relapse cases, treatment period and BMI (OR 4.71; 95%CI: 1.74,12.60; table 3).

**Table 3:** Analysis of patient characteristics to determine association with positive QuantiFERON-TB® Gold In-Tube result.

	Univariate Analysis			Multivariate Analysis, n = 100	
	n	Odds Ratio [95%CI]	p	Odds Ratio [95%CI]	p
CD4^+^≥200 cells/μl	105	4.71 [1.87,11.83]	0.001	4.68 [1.74,12.60]	0.002
Smear grade	111	1.28 [0.80,2.04]	0.297	1.12 [0.65,1.94]	0.691
Male sex	112	1.60 [0.67,3.78]	0.288	1.10 [0.29,3.07]	0.861
BMI<18.5	102	1.29 [0.53,3.18]	0.574	1.59 [0.58,4.38]	0.368
First TB episode	112	1.29 [0.44,3.73]	0.644	1.22 [0.33,4.46]	0.762
TB treated<2 weeks	111	1.17 [0.40,3.36]	0.777	1.71 [0.48,6.18]	0.408
Age>31 years	112	1.20 [0.51,2.81]	0.672	1.13 [0.41,3.10]	0.810

BMI, body mass index.

**Figure 2:**
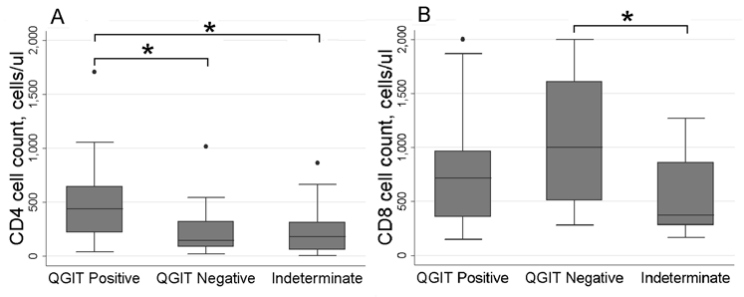
T lymphocyte counts by QuantiFERON-TB® Gold In-Tube result. A. Low CD4^+^ lymphocyte counts were associated with negative and indeterminate results whereas B. CD8^+^ lymphocyte counts were high in those with negative but low in those with indeterminate results. Box and whisker plots show range, inter-quartile range and median with dots representing outliers; QGIT, QuantiFERON-TB® Gold In-Tube; * significant difference, p<0.05 by Wilcoxon rank sum test.

Although a low CD4^+^ count was associated with both negative and indeterminate results (median 144, 181 cells/μl respectively; Wilcoxon p = 0.710), CD8^+^ count was high/normal in those with negative results but low in those with indeterminate results (median 999, 369 cells/μl respectively; Wilcoxon p = 0.017; figure 2).

### TST

**Figure 3:**
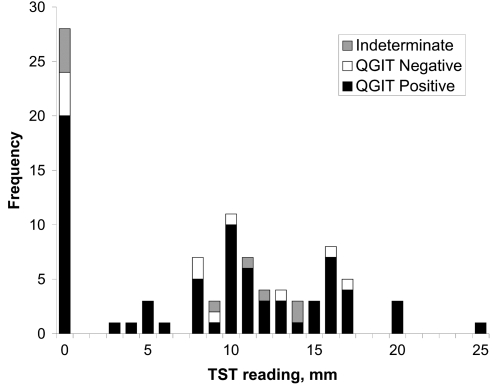
Frequency plot of tuberculin skin test readings showing QuantiFERON-TB® Gold In-Tube result. Concordance between TST and QGIT was poor with negative and indeterminate QGIT results occurring throughout the range of TST results. TST, tuberculin skin test; QGIT, QuantiFERON-TB® Gold In-Tube.

92 subjects had a TST result recorded (figure 3). 81/92 were recorded within the standard 48–72 hour period, 9/92 at 96 hours and 2/92 at 120 hours. Using a cut-off of 5 mm or more, 62/92 were TST positive, giving a sensitivity of 67% (95%CI: 58,77; table 4). There was no evidence of loss of TST sensitivity at >72 hours with 10/11 late readings being positive. 78 subjects had both known HIV status and a TST result. The test was impaired in HIV positive subjects with only 26/47 (55%) having a positive TST compared to 25/31 (81%) in the HIV negative group (Pearson's chi^2^, 1df, p = 0.021). A CD4^+^ count of less than 200 was clearly associated with negative TST (p<0.001). Presence of a BCG scar was not associated with variation in TST result (p = 0.264).

**Table 4:** Tuberculin skin test results showing both 5 mm and 10 mm cut-offs with subgroup analysis according to HIV status and CD4^+^ lymphocyte count and agreement with QuantiFERON-TB® Gold In-Tube.

		TST 5 mm cut-off, n/N_row_ (%)		TST 10 mm cut-off, n/N_row_ (%)	
		Positive	Negative	Positive	Negative
Overall		62/92 (67)	30/93 (33)	48/92 (52)	44/92 (48)
HIV Status	Positive	26/47 (55)	21/47 (45)	19/47 (40)	28/47 (60)
	Negative	25/31 (81)	6/31 (19)	19/31 (61)	12/31 (39)
	Unknown	11/14 (79)	3/14 (21)	10/14 (71)	4/14 (29)
CD4^+^, cells/μl	<200	9/28 (32)	19/28 (68)	6/28 (21)	22/28 (79)
	≥200	51/60 (85)	9/60 (15)	41/60 (68)	19/60 (32)
	Unknown	2/4 (50)	2/4 (50)	1/4 (25)	3/4 (75)
QGIT	Positive	51/73 (70)	22/73 (30)	40/73 (55)	33/73 (45)
	Non-positive†	11/19 (58)	8/19 (42)	8/19 (42)	11/19 (58)

QGIT, QuantiFERON-TB® Gold In-Tube; TST, tuberculin skin test.

† combined QGIT negative and QGIT indeterminate.

There was no significant difference between the overall sensitivities of TST and QGIT (McNemar's, p = 0.055). Although clearly affected by some of the same factors, agreement between the two tests was poor (agreement 64%, kappa 0.09; table 4). There was no improvement if only HIV negative subjects or those with CD4^+^ count>200 were included (agreement, kappa: 68%, −0.18; 77%, −0.01 respectively). Negative TST result was not particularly associated with either negative or indeterminate QGIT results (Pearson's chi^2^, 2df, p = 0.599; table 2).

Using a 10 mm cut-off, 48/92 were TST positive giving a reduced overall sensitivity of 52% (95%CI: 42,63; table 4). This sensitivity was significantly lower than QGIT's (McNemar's, p<0.001) and agreement remained poor (agreement 55%, kappa 0.08).

### Cut-off value for QGIT

Applying the cut-off suggested by Harada et al of ≥0.13 IU/ml to our data [21], 5 negative and 4 indeterminate results would have been reclassified as positive. All but one of these subjects were HIV positive. This adjustment would bring the overall sensitivity up to 82% (95% CI: 75,89). With the high background prevalence of LTBI in Zambia, it was deemed inappropriate to attempt to estimate IGRA specificity. Population specific ROC analysis could not be performed to determine the cost in terms of reduced specificity. No adjustment to the mitogen cut-off defining indeterminate results has been suggested.

## Discussion

This study investigated the performance of QGIT in a population with high TB incidence and HIV prevalence. As no gold standard exists for the diagnosis of LTBI, Pai et al recommend the use of microbiologically confirmed tuberculosis cases as an essential step in the assessment of new diagnostic tests [24]. This approach was taken, recruiting adult smear-positive TB patients into a cross-sectional study. QGIT was compared to TST as well as smear microscopy. The aim of the study was not to evaluate QGIT as a diagnostic test for active TB, but to increase understanding of the test's performance in this population by using active TB as a surrogate for LTBI.

The study estimates QGIT sensitivity at the lower end of the range of all other studies in active TB [13], but at a level similar to other studies in high burden settings [14], [15], [16]. As previously observed [14], [25], HIV infection was associated with decreased sensitivity with point estimates for HIV positive and negative groups being respectively well below and above the pooled sensitivity. As the sensitivity in the HIV negative patients was equivalent to those found in low burden countries, this study has shown that use of QGIT in the clinic setting in Zambia is technically possible, highlighting the utility of the portable incubator. There was, however, a significant change in the performance of the test in the population with CD4^+^ counts less than 100 cells/μl. Using QGIT to diagnose LTBI in Denmark and in San Francisco, the test was also found to perform poorly when CD4^+^ counts dropped below 100 cells/μl [5], [23].

It was expected that immunosuppression, whether due to severe TB or advanced HIV, would lead to a shift from QGIT positive to indeterminate results. Surprisingly, in the subgroups with lower QGIT sensitivity, an increase in not only indeterminate but also false-negative results was seen, with 20% of HIV positive subjects receiving a false-negative result.

It is possible that false-positive sputum smears would be associated with apparently false-negative QGIT. However the prevalence of non-tuberculous mycobacteria identified by sputum smear is thought to be very low, especially in high TB prevalence settings where specificity of a positive smear for MTB is excellent [26], [27]. A second explanation is that of specific anergy due to a predominance of regulatory TB specific T lymphocytes [28]. This would be unlikely to affect only HIV positive patients.

An alternative explanation is suggested by looking at the difference in CD4^+^ and CD8^+^ counts between those with negative and indeterminate results. Both CD4^+^ and CD8^+^ cells can respond to stimulation with phytoheamagglutin [29], used as the positive control in QGIT. However, because of their length, the overlapping peptides used as the TB test antigen are essentially MHC class II restricted and so only CD4^+^ cells respond. Subjects with low CD4^+^ in conjunction with high/normal CD8^+^ counts will therefore react to the positive control but not the test antigen and generate negative QGIT results, whereas suppression of both cell lines will lead to indeterminate results. As moderately advanced HIV is characterised by loss of number and function of CD4^+^ cells but intact antigen presenting and CD8^+^ cells [30], and TB is a feature of advancing HIV disease, this paradigm should be expected wherever TB and moderately advanced HIV are prevalent. An alternative positive test antigen targeting specifically CD4+ cells may eliminate this problem of false-negative results in such populations.

TST performed reasonably well overall but poorly in HIV positive subjects and especially in those with low CD4^+^ counts. Both QGIT positive-TST negative discordant results and a significant number of QGIT negative-TST positive results occurred. The interpretation of this is impossible within the limits of this study. The presence of typical BCG scarring did not seem to affect either test.

In their meta-analysis, Menzies and colleagues draw attention to the fact that all studies of QGIT to-date have used a cross-sectional design with many using active TB as a surrogate for LTBI [13]. Acknowledging practical, financial, and ethical reasons why this approach is so favoured, they emphasise that long-term prospective trials would be useful to inform the prognostic interpretation of the IGRA. That is not to say that the cross-sectional approach using active TB is not useful, but the results must be generalised with due caution.

As the test is measuring immunity, the diversity in the aetiology and natural history of disease states, both in terms of mycobacterial activity and the host's immune response, must be considered. There are clear changes between latent infection and active disease and differences between smear positive and smear negative disease. The transition from latent infection to active disease represents a break down in immune control, similarly smear negative disease is often associated with more extensive, less well contained infection than smear positive. Immunity is further impaired in severe TB possibly as cause or effect, but the patients in the majority of studies are ambulatory with mild to moderate disease. On a population level, ethnic differences in immune response and the effects and prevalence of immunomodulatory factors such as helminths and non-tuberculous mycobacteria are unclear [31]. The effect of TB treatment on the immune response is inconsistent although treatment up to one month was not seen to have any significant effect in the present study [16]. Interpreting these data must therefore be done with a clear idea of the population involved and their disease state. In the present study the subjects were similar in many ways to those in previous studies, they were adults with active, smear positive TB of mild to moderate severity. However, being set in Zambia, it is one of few studies in a sub Saharan African population with a high incidence of TB and high prevalence of HIV.

Although it is possible that false-positive smear results could lead to underestimation of QGIT sensitivity, using positive sputum smear as a gold standard probably overestimates the true overall sensitivity of QGIT in active TB. When mycobacterial culture was used in South Africa, sensitivity in smear negative disease was 65% compared to 80% in smear positive [14]. This is important as around two-thirds of patients treated in one of the clinics in Lusaka were registered with a smear negative diagnosis, the proportion being even higher in those with HIV (unpublished survey). As we know that active TB is associated with immunosuppression and develops via a breakdown of the immune control of latent infection, directly extrapolating the sensitivity in active disease to LTBI is conceptually challenging. If we assume that people with positive results in the active disease state would have had also been positive in the latent phase, the focus must be on understanding the causes of negative and indeterminate results.

### Conclusions

A tool to improve diagnosis of LTBI could be useful to direct isoniazid preventive therapy in high burden settings. Present barriers to the implementation of isoniazid preventative therapy policies are sited as cost, logistic problems and the difficulty of excluding active TB [8]. Introducing an IGRA would incur direct costs and also require considerable logistical and laboratory resources.

This study has shown that use of QGIT in the clinic setting in Zambia is technically possible. However, the accuracy of QGIT in populations with high prevalence of disease remains to be confirmed. A policy of providing isoniazid preventative therapy to all people living with HIV therefore continues to be an evidence based, safe, low-cost option [32]. Further research into IGRA in high burden settings is required both in terms of prognostic and programmatic issues.
